# Hearty Breakfasts

**Published:** 1875-08

**Authors:** 


### HEARTY BREAKFASTS.

In a large majority of cases it will be found that the best and heartiest meal of the day should be eaten in the morning. If the closing repast of the day has not been eaten too late, or has not been excessive in quantity or indigestible in quality, the stomach will be rested and active in the morning after the individual has enjoyed a cool bath. The stomach will then respond quickly with the necessary gastric juice for the solution of food, and, if a fair amount of exercise is taken during the day, a large mass of food will be assimilated and converted into blood and tissue. With a good, substantial breakfast no great amount of food will be required during the remainder of the day. One further meal will be ample, and that might better be taken at from two to three o'clock in the afternoon than at any later period, if business engagements only permitted it.

The breakfast may be made from any kind of wholesome food, and the fewer kinds the better. The dinner should be light and readily digested, if sound sleep is desired and a strong appetite and perfect powers of digestion next day. If hunger comes, a bowl of sweet milk and well-cooked mush of Indian meal, or other unbolted grain, will allay it, and will digest quickly. One "square meal" in every 24 hours is all that can be taken care of by many weak stomachs, and more than this is an excess and induces headache, nausea and distress. If dinners were abandoned, and especially late and heavy dinners, myriads of dyspeptics would be cured. But under the exigencies of city life a late dinner cannot well be avoided. This need not be the tremendous meal it is customary to make it, if the breakfast be substantial and nutritious, and not a thing of slops and biscuits, as it too often is.

---

The greater the knowledge, the greater the doubt.

### TO COOL A ROOM.

If the heat of a room occupied by an invalid is oppressive, it may be greatly lessened by hanging in the open windows some towels or canvas well wetted. Water, in passing from a liquid to a gaseous state, absorbs caloric. That chemical process will lower in a few minutes the temperature of a room by five or six degrees, and the humidity distributed in the air makes the heat more supportable. By that system the patients find themselves even in the height of summer, in an atmosphere refreshed, analogous to that which prevails after a storm.

---

A GOOD EDUCATION.—To read the English language well, to write with dispatch a neat, legible hand, and be master of the first four rules in arithmetic, so as to dispose of, at once, with accuracy, every question of figures which comes up in practice—we call this a good education. And if you add the ability to write pure grammatical English, we regard it an excellent education. These are the tools. You can do much with them, but you are helpless without them. They are the foundation; and unless you begin with these, all your flashy attainments, a little geology, and all other ologies and osophies, are ostentatious rubbish.

---

PURE OXYGEN IN PARALYSIS.—The other day M. Laurant, a prominent citizen, was struck with paralysis of the right side, and was unconscious, at Brussels. The physician called did not bleed, did not pour down drugs; he simply caused his patient to inhale pure oxygen for four hours. Then consciousness returned, and with it the power of motion and the sense of feeling. The next day the man was seemingly as well as ever. There is virtue in oxygen.

